# Nursing strategies after pancreaticoduodenectomy: continuity of care combined with immunomodulatory nutrition

**DOI:** 10.3389/fsurg.2025.1650974

**Published:** 2025-09-17

**Authors:** Xiaodong Feng, Wei Li, Yufu Tang, Lei Han, Ting Bi, Wei Zhang, Yuqing Miao

**Affiliations:** ^1^ Department of Hepatobiliary Pancreatic Spleen Thyroid Surgery, General Hospital of Northern Theater Command, Shenyang, Liaoning, China; ^2^ Department of Plastic Surgery, General Hospital of Northern Theater Command, Shenyang, Liaoning, China

**Keywords:** pancreaticoduodenectomy, continuity of care, immunomodulatory nutrition, postoperative recovery, quality of life

## Abstract

**Background:**

Pancreaticoduodenectomy (PD) is a highly invasive procedure with a long operation time, resulting in a high incidence of postoperative complications. The quality of patient care remains a key factor in improving prognosis. This study aimed to evaluate the impact of continuity of care combined with immunomodulatory nutrition on the postoperative recovery and quality of life of patients undergoing PD surgery.

**Methods:**

This was a randomized controlled study involving 120 patients who underwent PD surgery at our hospital between February 2022 and February 2024. The patients were randomly divided into the conventional care group (CG) and the intervention group (IG), with the latter receiving continuity of care combined with arginine-rich immunonutritional support. The main observation indicators included survival analysis, recovery of gastrointestinal function, length of hospital stay, nutritional status, immune function, postoperative complications, negative emotions and quality of life.

**Results:**

There was no significant difference in the overall survival rate between the two groups. Compared with the CG, the IG had shorter length of hospital stay, better recovery of gastrointestinal function, better improvements of nutritional indicators such as ALB and PAB, lower levels of pro-inflammatory factors TNF-α, IL-6 and CRP, better improvement of CD4^+^/CD8^+^ ratio, lower incidence of postoperative complications, lower SAS and SDS scores and better quality of life.

**Conclusion:**

Continuity of care combined with immunomodulatory nutrition provides a strong rationale for postoperative care of PD, and is worthy of clinical promotion and practice. More clinical cases and long-term follow-ups are needed to further verify its effectiveness.

## Introduction

Pancreaticoduodenectomy (PD) is currently the preferred surgical approach for treating pancreatic and pancreatic head cancers. Its technical maturity and clinical application value have been widely recognized. However, this surgery involves multiple organ resection and complex digestive tract reconstruction, and thus still has inherent characteristics such as significant surgical trauma and long operation time. It remains a highly challenging and complex surgical procedure in the field of surgery (1). Although the mortality rate of PD surgery has significantly decreased to 1.5%, with the advancement of surgical techniques, the incidence of postoperative complications remains high. Statistical data show that it fluctuates between 20% and 60% (2, 3). This contradiction between the quality of postoperative recovery and the safety of the surgery has prompted clinical research to continuously explore optimization strategies.

The current research mainly focuses on two aspects: one is the innovation of surgical techniques, such as robotic-assisted resection which reduces tissue damage through precise operation, and the improved PD which lowers the risk of pancreatic fistula by optimizing the anastomosis method (4, 5). Secondly, there has been an innovation in the nursing model. Researchers such as Liu et al. implemented a personalized pathological management nursing model for patients undergoing complex abdominal surgeries. Through a three-month follow-up, it was confirmed that this model significantly improved the recovery quality of the patients (6). These explorations all indicate that optimizing perioperative management plays a crucial role in improving the prognosis of PD patients. However, most existing studies focus on the effect evaluation of single intervention measures, lacking systematic research on comprehensive intervention models.

The continuity of care model, as a new type of nursing model, extends professional nursing services from hospitals to homes and communities, thereby establishing a continuous and coordinated health management system (7). This model has been preliminarily verified to have advantages in the fields of prostate cancer (8), obstetric diseases (9) and mental disorders (10), but its application in patients undergoing major abdominal surgeries still lacks high-quality evidence support. It is particularly noteworthy that the gastrointestinal dysfunction caused by PD surgery often leads to a progressive decline in lean body mass (LBM), and this metabolic change is closely related to immune function suppression and an increased risk of infection (11). Although immune nutritional support, by supplementing immune-regulating components such as arginine and *ω*-3 polyunsaturated fatty acids, has been proven to effectively alleviate postoperative inflammatory responses and enhance immune function (12). However, at present, there is still a lack of research on the synergistic effect of continuity of care and immunonutrition.

Based on this, this study intended to establish a comprehensive intervention model combining continuity of care and immunonutrition, with the main focus on exploring its impact on the postoperative recovery quality, complication occurrence rate, and long-term survival status of PD patients. The aim was to provide new theoretical basis and practical solutions for optimizing the perioperative management of major abdominal surgeries.

## Materials and methods

### Study design

This was a randomized controlled study aimed at investigating the postoperative care strategies for patients undergoing PD, evaluating the postoperative recovery and quality of life of patients who received nutritional support rich in arginine combined with continuity of care, and providing pilot research and data support for the referral strategies of postoperative care for PD.

### Participants

One hundred and twenty patients who underwent PD in our hospital from February 2022 to February 2024 were selected as study subjects. Inclusion criteria: (1) Met the relevant indications for PD; (2) Had no previous history of upper abdominal surgery; (3) Received postoperative continuity of care. Exclusion criteria: (1) The lesion had already metastasized to distant sites and could not be cured by surgical resection; (2) Had severe organic diseases; (3) Had mental disorders or cognitive impairments; (4) Those with other types of malignant tumors combined. The study was approved by the Ethics Committee of the hospital, and all subjects signed the informed consent form before enrollment.

### Randomization and blinding

**Figure 1:**
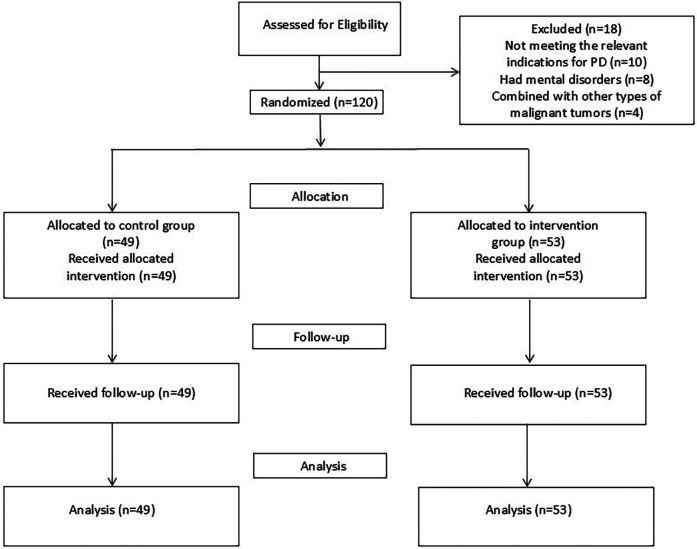
CONSORT flowchart.

A group randomization design was adopted for random grouping. The random allocation sequence was generated by a computer. The allocation confidentiality measures were achieved through sequential numbering, sealing, and opaque envelopes. After being deemed to meet the inclusion criteria, patients were randomly assigned to the control group (CG) or the intervention group (IG), as shown in Figure 1. This study was single-blind, and the participants were unaware of the allocation.

### Sample size calculation

Power analysis was carried out in this study using G*Power 3.1.9.7 software to determine the sample size required to detect statistical differences. With an alpha level of 0.05% and 90% power analysis, the research revealed that a sample size of 48 patients per group was required.

### Intervention

The CG was given routine care, including close monitoring of the patients’ baseline vital signs after the operation, providing nutritional feeding 4 hours after the operation to supply nutrients (the same as IG, Table 1), psychological intervention, rehabilitation exercises, routine health education and other guidance. The patients needed to undergo regular re-examinations every two months to understand their recovery status.

**Table 1:** Nutritional formulations.

Formulas	IG	CG	Feeding methods
Energy	1,250 kcal	1,250 kcal	The feeding rate was 25 ml/hour after 4 h postoperatively; from 24 h-48 h postoperatively, the feeding mode was changed from jejunostomy feeding to transoral feeding by infusion pump, and the feeding rate was 50 ml/hour; from 48 h-10 d postoperatively, the feeding rate was 75 ml/hour per day, with a 4 h rest period every day.
Total protein	75 g	75 g	
Total fat	42 g	42 g	
Triglyceride	45 g	45 g	
*ω*6:ω3 fatty acid ratio	3.5:1	5:1	
Carbohydrate	140 g	130 g	
K+	55 mmol	45 mmol	
Na+	50 mmol	35 mmol	
Osmolalilty (mOsm/kg)	380	270	
Arginine	9.0 g	3.0 g	
Glutamine	13 g	7.5 g	
Cysteine	0.7 g	0.2 g	

The IG was given continuity of care combined with immunomodulatory nutritional support. The specific methods were as follows:

1. Establishment and training of the nursing team: The head nurse served as the team leader, responsible for organizing the training of team members and supervising the quality of nursing services. This department consisted of 10 experienced nurses with over 3 years of on-the-job experience, who formed the team and were responsible for the implementation of specific nursing operations. The head nurse of the hospital organized the training of team members, including professional knowledge and nursing technology guidance after PD, formulating unified nursing standards, and passing the pre-service examination before officially carrying out nursing work.
2. Implementing continuity of care: a. Immunomodulatory nutrition: The patient began enteral feeding through the jejunum 4 hours after the surgery. The feeding formula and feeding rate were as described previously, as seen in Table 1. b. Pain care: if the patient could tolerate the current pain, the nurse adjusted the patient's emotional state according to the patient's interests, such as through watching TV, listening to music, and diverting attention to alleviate the pain. For those patients who could not tolerate the current pain, the nurse administered pain-relieving medication to alleviate the pain. After the surgery, patients often worry about the treatment outcome and prognosis. The nursing team used this as a starting point for psychological care, answering patients’ questions and resolving their psychological issues, striving to alleviate the patients’ negative psychological reactions, so that they could overcome the disease with a good mindset. c. Exercise guidance: After the patient regained consciousness, the nurse instructed him/her to carry out ankle pump exercise on the bed. The specific operation method was as follows: The patient lied on their back, with both legs straightened, the thighs relaxed, the calves tightened, and tried to lift the toes as high as possible to the maximum angle. After the patient reached the maximum angle of lifting the toes upward, forcefully pushed them downward and kept this position for 5 s. This action was performed 5 times. As a set, the nurse suggested that the patient performed this exercise for 2 minutes every hour to prevent thrombosis. After returning to the general ward, the nurse guided the patient to carry out lower limb activities based on their specific condition. d. Health education: The healthcare staff invited patients or family members to join the continuity of care WeChat group. The healthcare staff used the health education resources of the medical association and regularly released the knowledge of post-PD care through WeChat groups (including text, pictures and videos), in order to enhance the nursing knowledge level of patients and their families. e. Follow-up: After the patient was discharged, the nursing staff carried out a telephone follow-up once a week to understand the patient's recovery status. They conducted a monthly follow-up to guide the patient to return to the hospital for re-examination on time and to promptly return to the hospital for re-examination in case of any abnormal conditions. The nursing plan was adjusted based on factors such as the patency of the drainage tube and the patient's nutritional status.

Both groups were intervened for 6 months.

### Primary outcomes

1. Survival analysis. The overall survival within six months of care was recorded.
2. Gastrointestinal function recovery. The time of first anal defecation, time of first meal, time of bowel sound recovery, and time of first bowel movement were counted in the two groups respectively.
3. Hospitalisation time. Ventilator use time, ICU hospitalisation time, time of getting out of bed and hospitalisation time of the two groups were counted respectively.
4. Nutritional status. Fasting venous blood was collected before, 3 d, 7 d and 14 d after the nursing care, and the levels of ALB, PAB, TP and TF were measured by automatic biochemical analyser (AU480, Beckman, USA).
5. Immune function indicators. Fasting venous blood was collected before, 3 d, 7 d and 14 d after the nursing care, and the levels of TNF-α, IL-6 and CRP were measured by automatic biochemical analyser, and the levels of CD4 + and CD8 + were detected by flow cytometer (EPIC, Beckman, USA), and CD4+/CD8 + were calculated.
6. Postoperative complications. The incidence of pancreatic fistula, anastomotic bleeding, gastroparesis, incision infection, and lower limb venous thrombosis in the six months after the patients were discharged from the hospital was counted.

### Secondary outcomes

1. Negative emotions. The SAS and SDS scales were completed before, after, and six months after the patients were discharged from the hospital. The SAS scale assessed the patients’ anxiety level, and the SDS assessed the patients’ depression level. The higher the score, the more severe the degree of anxiety and depression.
2. Quality of life. A comprehensive quality of life assessment questionnaire (GQOLI-74) was used to comprehensively assess the quality of life of the two groups after surgery, the scale contains four dimensions such as somatic function, psychological function, social function, and material life status. The scores of the first three dimensions were 20–100 points, and the score of the fourth dimension was 16–80 points, and the higher the score indicated that the patient's quality of life after surgery was better.

### Statistical analysis

In order to test whether there was any difference in the variables between the groups, quantitative analyses were carried out using the *χ*2 test in SPSS 21.0 according to the test conditions. All normally distributed continuous data were analysed using Student's *t*-test while non-parametric data were analysed using Mann—Whitney *U*-test. Cumulative survival probabilities were estimated using the Kaplan–Meier method and the significance of differences between survival rates was tested using the log-rank test (Log-ranking). Count data and measurements are expressed as *n* (%) and mean ± SD, respectively, and all tests were two-sided. *p* < 0.05 was considered a statistically significant difference.

## Results

A total of 120 patients who underwent PD were collected in this study, of which CG was shed in another 11 cases and IG was shed in 7 cases. The mean age of the patients was (60.11 ± 9.6) years, the maximum age was 79 years and the minimum age was 28 years, and the most common type of pathology was pancreatic cancer. There was no statistical difference between the baseline data of the two groups (*P* > 0.05, Table 2).

**Table 2:** Baseline characteristics of patients (mean ± standard deviation) or *n* (%).

Parameter		CG	IG	*X^2^ (t)*	*P*
Number		49	53	–	–
Age (years)		59.71 ± 8.98	60.65 ± 9.77	0.549	0.584
Place of residence, *n* (%)	Town	42 (70.00)	37 (61.67)	0.926	0.336
	Village	18 (30.00)	23 (38.33)		
Gender, *n* (%)	Female	21 (35.00)	25 (41.67)	0.564	0.453
	Male	39 (65.00)	35 (58.33)		
BMI (kg/m^2^)		22.07 ± 2.71	22.15 ± 2.59	0.163	0.869
Medical history, *n* (%)	Hypertensive	36 (60.00)	34 (56.67)	0.187	0.980
	Diabetes	21 (35.00)	22 (36.67)		
	Hepatitis B	23 (38.33)	24 (40.00)		
	Fatty liver	18 (30.00)	20 (33.33)		
Pathological type	Pancreatic cancer	22 (36.67)	29 (48.33)	1.726	0.631
	Carcinoma of the bile ducts	17 (28.33)	19 (31.67)		
	Duodenal papilloma	12 (20.00)	10 (16.67)		
	Carcinoma of the ampulla oblongata	9 (15.00)	6 (10.00)		

### Survival analysis

**Figure 2:**
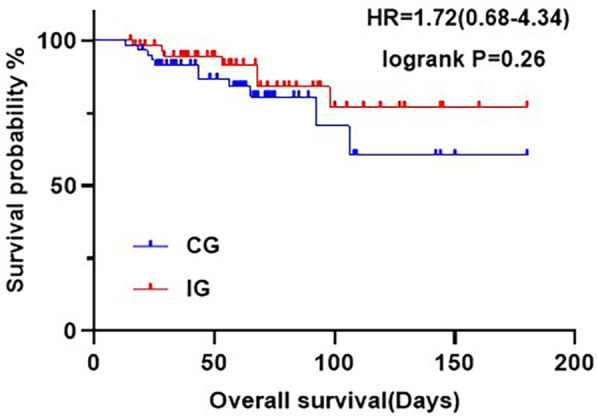
Kaplan-Meier curves showing OS at six months postoperatively for patients in the different care groups.

At six months, there was no statistically significant difference in overall survival rates between the two groups (*P* = 0.25), The survival rate of the CG was 81.67%, while that of IG was 88.33% (Figure 2).

### Assessment of gastrointestinal function

**Figure 3:**
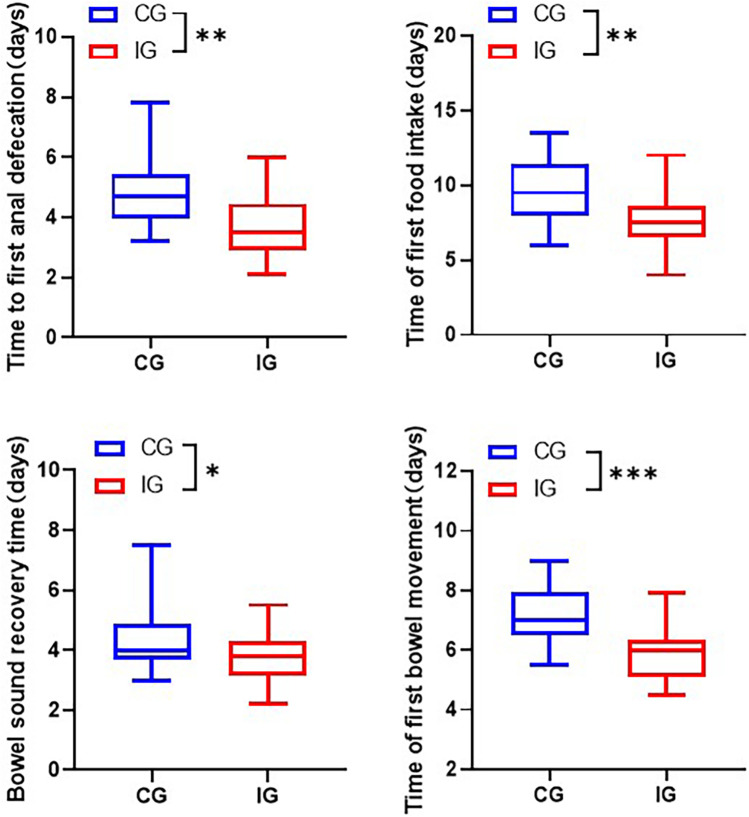
Assessment of gastrointestinal indicators in patients of different care groups. *p < 0.05, **p < 0.01, ***p < 0.001.

Compared with the CG, the gastrointestinal function indexes of the IG showed significant improvement. Specifically, the time to first anal defecation shortened from (4.92 ± 1.25) days to (3.73 ± 1.09) days, the time to first feeding shortened from (9.76 ± 2) days to (7.82 ± 1.89) days, the time to recovery of bowel sounds shortened from (4.37 ± 1.02) days to (3.75 ± 0.85) days, and the time to first defecation shortened from (7.21 ± 1) days to (5.92 ± 0.94) days (Figure 3).

### Comparison of length of hospitalization

**Figure 4:**
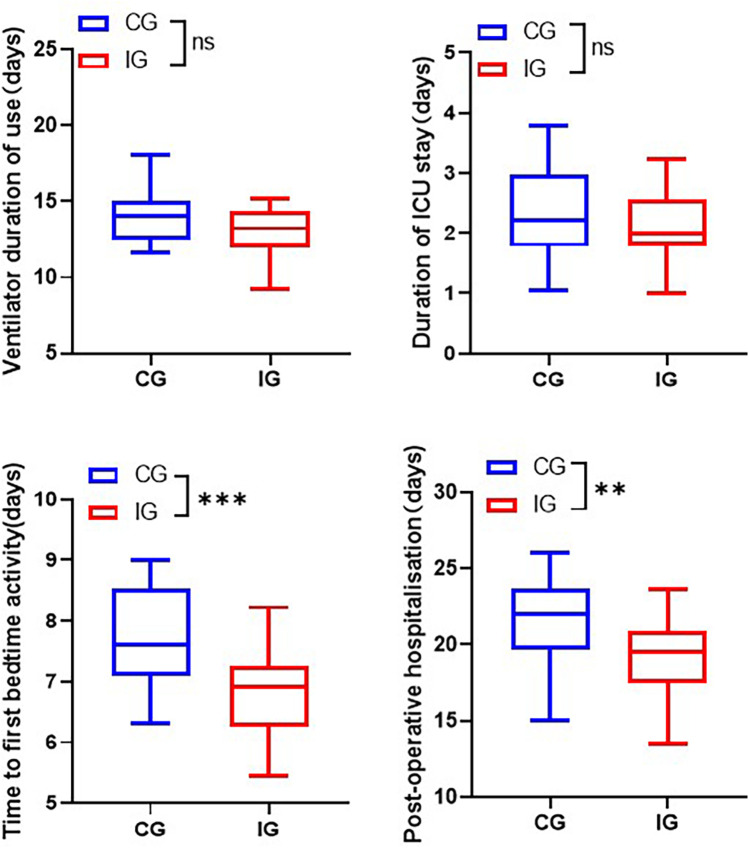
Comparison of length of stay of patients in different care groups. **p < 0.01, ***p < 0.001.

The total hospitalization time for patients in the IG was shorter than that of the CG. Specifically, the duration of ventilator use reduced from (14.06 ± 1.82) days to (13.04 ± 1.56) days, the length of stay in ICU shortened from (2.32 ± 0.78) days to (6.77 ± 0.70) days, and postoperative stay reduced from (21.61 ± 2.75) days to (19.17 ± 2.58) days (Figure 4).

### Assessment of nutritional status

**Figure 5:**
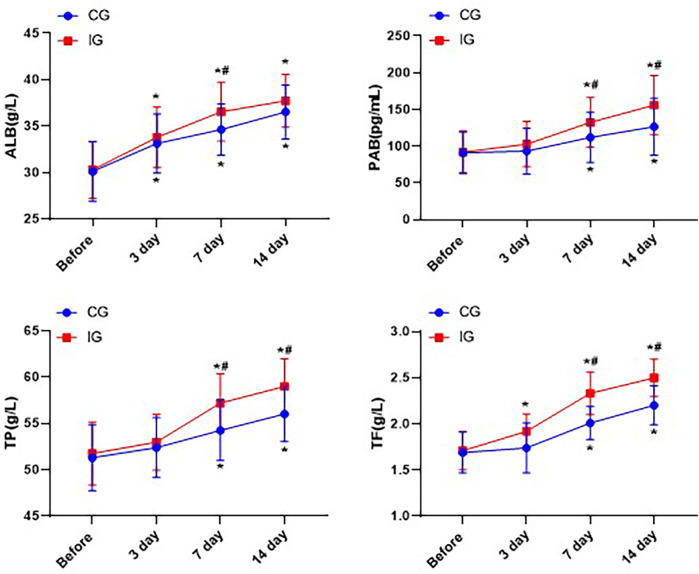
Comparison of nutritional indicators of patients in different nursing groups. *P < 0.05, *compared with before nursing. #*P* < 0.05, # compared with CG.

Within 14 days after the treatment, the levels of ALB, PAB, TP and TF in both groups of patients significantly increased, and the increase in each nutritional index was significantly greater in the IG than in the CG (*P* < 0.05, Figure 5).

### Assessment of indicators of immune function

**Figure 6:**
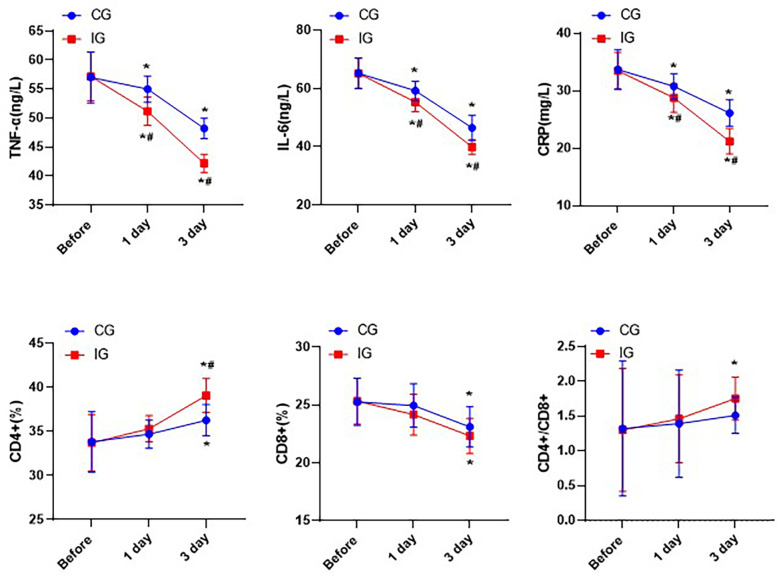
Comparison of immune function indexes of patients in different nursing groups. *P < 0.05,*compared with before nursing. #*P* < 0.05, # compared with CG.

The levels of TNF-α, IL-6 and CRP in both groups showed a downward trend, while the proportion of CD4 + cells increased, the proportion of CD8 + cells decreased, and the ratio of CD4+/CD8 + increased. In addition, one day after treatment, the levels of pro-inflammatory factors in the IG were significantly lower than those in the CG. Three days after treatment, the proportion of CD4 + cells in the IG was significantly higher than that in the CG, indicating that the immune function of the IG was better than that of the CG (*P* < 0.05, Figure 6).

### Evaluation of postoperative complications

Compared with the CG, the postoperative incidence rates of pancreatic fistula, anastomotic bleeding, gastroparesis, incision infection, and lower extremity venous thrombosis in the IG was lower. There was a statistically significant difference in the incidence of lower extremity venous thrombosis between the two groups (*P* < 0.05, Table 3).

**Table 3:** Complication statistics for both groups of patients, *n* (%).

Groups	Pancreatic fistula	Anastomotic bleeding	Gastrop-aresis	Cutaneous infection	Lower limb venous thrombosis
CG (*n* = 49)	4 (8.16)	1 (2.04)	9 (18.37)	2 (4.08)	11 (22.45)
IG (*n* = 53)	2 (3.77)	0 (0.00)	4 (7.55)	1 (1.89)	4 (7.55)
X^2^	0.886	1.01	2.68	0.43	4.508
P	0.347	0.315	0.102	0.512	0.033*

* *P* < 0.05.

### Assessment of negative emotions

**Figure 7:**
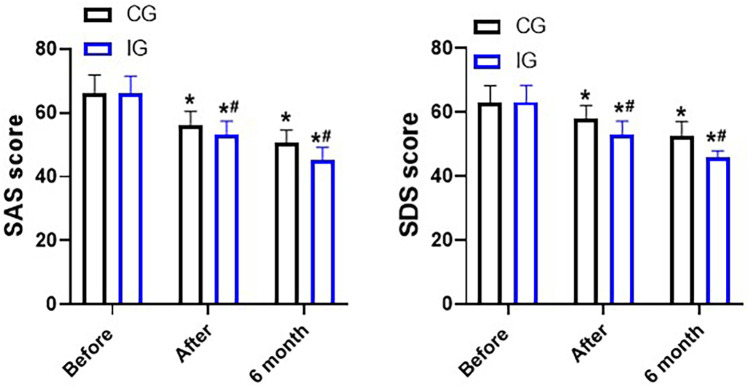
SDS and SAS scores, points. *P < 0.05, *compared with before nursing. #*P* < 0.05, # compared with CG.

The SAS scores and SDS scores of patients in both groups decreased significantly after the nursing care. Moreover, the SAS scores (45.27 ± 0.85) and SDS scores (45.27 ± 0.85) of the IG were within the normal range after six months, which indicated that continuity of care combined with immunomodulatory nutrition could better alleviate the anxiety and depression of the patients within six months (Figure 7).

### Assessment of quality of life

**Figure 8:**
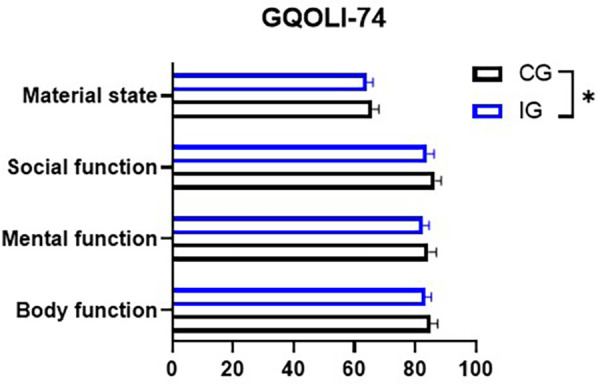
GQOLI-74 assessment of postoperative quality of life. *P < 0.05.

In both groups of patients, after receiving the nursing treatment, their physical functions, psychological functions, social functions, and living material conditions all showed significant improvements. Among them, the improvement of the patients in the IG was more obvious (*P* < 0.05, Figure 8).

## Discussion

PD have traumatic characteristics, so corresponding care is needed during the postoperative stage to improve the quality of life of the patients. PD is a major surgery, and the patients’ preoperative conditions are relatively severe, usually accompanied by malnutrition and low immune function. Coupled with the effects of intraoperative stress and postoperative inflammatory responses, it may lead to a series of postoperative complications, which will further affect their postoperative recovery (2, 13). Therefore, this study aimed to investigate the impacts of effective postoperative care on the prognosis and quality of life of patients undergoing PD.

Malnutrition is one of the main causes leading to an increase in postoperative complications and mortality among patients (14). Enteral nutrition can provide easily digestible and absorbable nutrients, reduce the burden on the pancreas and liver, and protect the functions of body organs (15). At the same time, it can stimulate the secretion of insulin, gastrointestinal hormones and other hormones, regulate blood glucose and gastrointestinal tract function, helping to restore the nutritional state of the patients and improve various indicators related to nutrition (16). In addition, it can maintain the microecological balance of the intestine, promote the growth and reproduction of beneficial bacteria, inhibit the growth and reproduction of harmful bacteria, and further reduce the inflammatory responses (17). Moreover, enteral nutrients can also regulate the acid-base balance in the gastrointestinal tract, reduce gastric acid secretion, alleviate the damage caused by gastric acid, and help maintain the normal function of the gastrointestinal tract (18, 19). The research has found that gradually providing enteral nutrition support to patients in the early postoperative period (within 24 hours) does not increase the incidence of adverse gastrointestinal reactions, and this approach is feasible (20). Adiamah et al. revealed that administration of enteral nutrition at 4 h postoperatively had no adverse effect on the postoperative nutritional recovery of patients (21). Therefore, we provided enteral nutrition support within 4 h after the operation, and started oral feeding after 24 h after the operation.

Immunonutrition is a nutritional formula containing a variety of amino acids, fatty acids, while our formula mainly increased the content of arginine and glutamine. The research has found that arginine can significantly increase the albumin level and lymphocyte count of cancer patients, reduce the incidence of postoperative infections, and shorten the hospital stay (22). Meanwhile, glutamine has the effects of improving immune function, promoting protein synthesis, and protecting intestinal function (23).

In our study, we found that the gastrointestinal function of the IG was significantly improved, as evidenced by the significant shortening of the first defecation time, the first defecation interval, and the first eating time. The findings indicated that the imbalance of the intestinal physiological environment was alleviated, and the probiotics in the intestinal tract were up-regulated. In addition, nutritional indicators of the IG, such as ALB, PAB, TP and TF, all showed significant improvement within 14 days after treatment. This was consistent with the findings of Abreu et al. (24), which stated that immune nutritional substances could enhance the host's immune function and regulate the intestinal flora and homeostasis.

The immune status of the human body plays an important role in the progression and prognosis of patients with malignant diseases, and is also a key factor affecting the recurrence and metastasis of patients after surgery (25). Traumatic stress is one of the causes leading to the decline of the body's immune function (26). Humoral immunity mediated by B lymphocytes and cellular immunity mediated by T lymphocytes are important components of the immune system in the human body (27). In cellular immunity, T lymphocytes, as a group of multifunctional immune response cells, play roles in immune surveillance and target cell killing (28). Their subsets, CD4 + and CD8+, participate in immune responses and exert cytotoxic effects in eliminating tumor cells. When CD4 + is activated by the corresponding tumor antigen, it can secrete various cytokines to exert anti-tumor effects. On the other hand, CD8 + has cytotoxicity and can kill tumor cells (29). When the CD4+/CD8 + ratio is abnormal, it indicates that the immune status has been damaged, which may cause tumors and autoimmune diseases (30). One day after the surgery, the CD4 + ratio in the IG significantly increased, while the CD8 + ratio decreased significantly. Meanwhile, the ratio of CD4+/CD8 + gradually returned to the normal range, indicating that immune nutrition could improve the immune function of patients and activate the immune response to exert a killing effect. Surgical trauma induces the body to produce pro-inflammatory factors such as TNF-α and IL-6. These pro-inflammatory factors exacerbate the inflammatory response by stimulating the inflammatory cells in the body, thereby affecting the postoperative recovery of the patients (31). In this study, the levels of pro-inflammatory factors TNF-α, IL-6 and CRP decreased significantly after 3 days of care. We initially hypothesized that this might be due to the improvement in nutritional status and immune function. Additionally, the surgical trauma itself does not cause the body to secrete a large amount of pain factors, thereby leading to severe pain (32). Postoperative pain can cause stress and anxiety for patients, and increase the likelihood of delayed complications during the recovery of gastrointestinal function after surgery (33). Therefore, reasonable pain management is also an important part of the nursing work. Standardized assessment of patients’ pain and reasonable management can help relieve patients’ pain and negative emotions, and can also help patients get out of bed and perform functional exercises as soon as possible after the surgery, promoting intestinal peristalsis, and accelerating the circulation of the whole body and the circulation of the gastrointestinal tract, thereby promoting the recovery of gastrointestinal function. Compared with the CG, the SAS and SDS scores as well as the gastrointestinal function of the IG were significantly improved. This may be attributed not only to the reasonable management of pain, but also to the significant improvement of the patient's clinical symptoms, thereby reducing anxiety and depression.

For the elderly, due to changes in vascular conditions and the presence of diseases such as tumors, the blood becomes in a hypercoagulable state. The overall incidence of lower limb venous thrombosis in hospitalized patients is approximately 1%, and in postoperative abdominal surgery patients it is approximately 20%, while the incidence among postoperative abdominal surgery patients is about 20%. Moreover, prolonged surgical procedures will further increase the incidence of venous thrombosis (34, 35). During the period when the patients were bedridden, the nurses instructed and supervised them to perform ankle pumping exercises for both lower limbs. Reasonable guidance for patients to get out of bed for activities can, to a certain extent, prevent the occurrence of lower limb venous thrombosis. In this study, the incidence of lower limb venous thrombosis in the IG (7.55%) was significantly lower than that in the CG (22.45%). After undergoing PD, pancreatic cancer patients have a higher risk of recurrence and metastasis, so they need continuous and coordinated care to avoid deterioration of the condition. Pancreatic cancer patients and their families need to understand and master the knowledge of postoperative rehabilitation. In the case of a lower survival rate, more emphasis should be placed on improving the effectiveness of survival treatment and relying on the original guidance to meet the needs of patients. Finally, this study analyzed the survival rate of patients six months after surgery, and the statistical results showed no statistical difference between the two groups of patients.

### Limitations

Our research has some limitations. Firstly, our sample size is relatively small, which may lead to deviations between the data results and the actual values. Secondly, our research adopted a single-blind design, which inevitably resulted in subjective biases from the researchers, leading to an imbalance in the treatment between the two groups. Thirdly, our research was a single-center study, and the sample was not representative, which may not accurately reflect the characteristics of a broader population. Fourthly, the six-month follow-up period is too short. The effects of continuity of care combined with immunomodulatory nutrition on the long-term prognosis of patients undergoing PD are currently unclear. Therefore, more multi-center, double-blind, large-scale, and long-term studies should be conducted in the future to further verify our findings.

## Conclusion

In this study, the postoperative care of patients undergoing PD was optimized, and multidimensional care was provided in aspects such as pain care, psychological care, exercise care, health education and immuno-nutrition. This resulted in significant improvements in the immune function, gastrointestinal function, nutritional level, and negative mood of the patients, and a certain reduction in the postoperative complications, thereby enhancing the patients’ quality of life. However, this model of care had no significant effect on the mortality rate of patients within six months. Therefore, it is necessary to collect more clinical cases and extend the follow-up period to further study. In conclusion, from this study, we found that continuity of care combined with immunomodulatory nutrition is an effective nursing model, which provides a strong basis for subsequent nursing care and is worthy of promotion and application in clinical practice.

## Data Availability

The original contributions presented in the study are included in the article/Supplementary Material, further inquiries can be directed to the corresponding author.
